# Distribution and Determinants of Peripapillary Retinal Nerve Fiber Layer Thickness and Its Association with Sleep Quality in Chinese Teenagers

**DOI:** 10.1155/2019/6510203

**Published:** 2019-09-05

**Authors:** Chen-Wei Pan, Yu-Xi Qian, Hua Zhong, Jun Li, Qin Chen

**Affiliations:** ^1^School of Public Health, Medical College of Soochow University, Suzhou, China; ^2^Department of Ophthalmology, The First Affiliated Hospital of Kunming Medical University, Kunming, China; ^3^Department of Ophthalmology, The Second People's Hospital of Yunnan Province, Kunming, China; ^4^Department of Ophthalmology, The First Affiliated Hospital with Nanjing Medical University, Nanjing, China

## Abstract

**Purpose:**

We aimed to evaluate the distribution and determinants of peripapillary retinal nerve fiber layer (pRNFL) thickness and its associations with general sleep quality in Chinese school students.

**Methods:**

1063 grade 7 students aged 13 to 14 years with pRNFL thickness data from a school-based study on grade 7 students in Southwestern China participated in the study. The pRNFL thickness was measured on the optical coherence tomography images of a circular scan centered on the optic disc. Refractive error was measured after cycloplegia using an autorefractor and biometric parameters including axial length (AL) were measured by an IOLMaster. Participants' sleep quality was measured by the Children's Sleep Habits Questionnaire (CSHQ).

**Results:**

The mean pRNFL thickness was 106.8 ± 10.7 *μ*m among the 1063 participants. There was an increasing trend of spherical equivalent and a decreasing trend of AL with RNFL thickness. In multivariate analysis, each diopter of spherical equivalent increase was associated with 0.64 *μ*m increase in pRNFL thickness. Girls had an increased mean pRNFL thickness compared with boys with a mean difference of 1.65 *μ*m. Per 10 *μ*m increase in pRNFL thickness was significantly associated with a 0.5 reduction in CSHQ score (better sleep quality).

**Conclusions:**

More myopic refractive error was the major ocular determinant of decreased pRNFL thickness. In addition, students with thinner pRNFL tended to have a worse sleep quality.

## 1. Introduction

Clinically, the peripapillary retinal nerve fiber layer (pRNFL) thickness is an important ocular parameter in the diagnosis and monitoring of glaucoma [1], which is a leading cause of irreversible vision loss and blindness [2]. In addition to glaucoma, pRNFL thickness plays a role to aid the diagnosis of systemic nerve diseases such as dementia [3], stroke [4], and multiple sclerosis [5]. Thus, understanding the normative distribution of pRNFL thickness and its predictors in general population is crucial in the clinical management of these ocular and systemic nerve diseases.

With rapid development of new ocular imaging technologies, spectral-domain optical coherence tomography (SD-OCT) has been widely used in the assessment of pRNFL thickness in clinical practice. Compared with traditional methods such as ophthalmoscopy and fundus photography, SD-OCT has a higher repeatability and reproducibility in the assessment of the pRNFL thickness [6]. By using SD-OCT, several epidemiologic studies have reported the distribution and determinants of pRNFL thickness in middle-aged to elderly adults [7–12]. Some data on younger generations have been reported in hospital-based clinical studies with small sample size and thus were susceptible to selection bias [13–15]. In fact, the assessment of pRNFL thickness also has important implications in the diagnosis of several pediatric nerve disorders such as congenital glaucoma [16] and Marfan syndrome [17].

In addition, there is sufficient evidence suggesting that pRNFL thinning might be a window for detecting sleep disorders such as obstructive sleep apnea syndrome with thinner pRNFLs being related to a higher risk of obstructive sleep apnea syndrome [18]. Obstructive sleep apnea syndrome is prevalent in both adults and children and is often linked with a reduced sleep quality [19]. However, few studies have assessed the associations between pRNFL thickness and general sleep quality, especially in younger generations. Assessing the relationship between pRNFL thickness and general sleep quality may provide novel clues in understanding the intrinsic link of the eye with circadian rhythm of the whole body. Our hypothesis is that pRNFL thinning might be an ocular marker for reduced sleep quality among adolescents.

The current study had two aims. First, we aimed to evaluate the distribution and determinants of mean pRNFL thickness measured by SD-OCT in a general population of rural Chinese adolescents. In addition, we aimed to assess the association between pRNFL thickness and general sleep quality as measured by the Children's Sleep Habits Questionnaire (CSHQ).

## 2. Materials and Methods

### 2.1. Study Population

The data of the analysis were from the Mojiang Myopia Progression Study, which is a school-based study performed in Mojiang located in the southwestern part of China. Detailed study protocol and some significant findings have been reported in previous publications [20–24]. In short, the original study cohorts included elementary school grade 1 and middle-school grade 7 students. The baseline survey included the students who were considered to be eligible to participate in the study, that is, he or she should have been living in Mojiang for at least one year and planned to live there for at least four years. We invited the students and their parents to participate in the study through cell phone messages. For those who did not agree to participate or did not respond, telephone interview was made to let them better understand the nature of the study. If the parents could not be reached by cell phone message or telephone, home visits were made. Ethics committee approval was obtained from the Institutional Review Board of Kunming Medical University. The study was conducted according to the tenets of the Declaration of Helsinki involving human participants and the approved guidelines. We obtained written informed consents from at least one parent or legal guardian of each participant.

### 2.2. RNFL Thickness Measurement

Due to limited resources in manpower, pRNFL thickness measurement was only performed in selected grade 7 students. In each school, we randomly selected two classes and all the students in the selected classes were invited to participate in the SD-OCT measurement. Among all the 2346 grade 7 students aged 13 to 14 years, pRNFL thickness data were obtained from 1063 students. SD-OCT (Heidelberg Engineering Co., Heidelberg, Germany) was used to assess the optic nerve head of the selected participants. The software of the OCT device was used to measure the pRNFL thickness on a circular scan centered on the optic disc. The diameter of the circular scan was set at 3.4 mm. Automatic segmentations were performed by the OCT software and the segmentations were checked and adjusted manually when necessary. The pRNFL measurement circle was manually selected in 62 participants, among whom only 3 were highly myopic.

### 2.3. Sleep Quality Assessment

The CSHQ was used to assess the general sleep quality [25]. The CSHQ was completed by parents asking about children's sleep habits in a typical recent week. The Chinese version of CSHQ has been validated in a previous report [26], which included eight various domains such as bedtime resistance, sleep onset delay, sleep duration, sleep anxiety, night waking, parasomnias, sleep-disordered breathing, and daytime sleepiness [25]. Each item was scored on a 3-point scale: rarely, 0 to 1 time per week; sometimes, 2 to 4 times per week; and usually, 5 to 7 times per week.

### 2.4. Other Measurements

Refractive error of both eyes was measured after cycloplegia using an autorefractor (RM-8000; Topcon Corp., Tokyo, Japan). Spherical equivalent (SE) was defined as sphere plus half cylinder. An IOLMaster (Carl Zeiss Meditec AG, Jena, Germany) was used to measure ocular biometric parameters including axial length (AL), anterior chamber depth (ACD), and corneal power (CP). Intraocular pressure (IOP) was measured using a handheld tonometer (TONO-PEN AVIA; Reichert Inc., NY, USA). For the measurement of ocular biometric parameters and IOP, three repeated readings were obtained and averaged before cycloplegia. Height was measured to the nearest 0.1 cm using a wall-mounted audiometer. Participants stood straight, barefoot, with relaxed shoulders and their arms hanging freely. Weight was measured to the nearest 0.1 kg using a scale, with minimal clothing and without shoes. Body mass index (BMI) was calculated as the weight divided by the square of the height. For measuring blood pressure (BP), participants sat in a chair for five minutes of rest, while the right arm was supported at the heart level. BP was measured with a mercury column sphygmomanometer and then the 1st and 5th Korotkoff sounds were used to determine the systolic and diastolic BP. Information regarding birth weight was retrieved from the participants' delivery records.

### 2.5. Statistical Analysis

The statistical analysis was performed using STATA version 11.0 (StataCorp, College Station, Tex., USA). Descriptive statistics of the variables including the means, standard deviations, and percentages were presented where appropriate. As the measurement of pRNFL thickness was highly correlated between the two eyes, only the data of right eye were included in the analysis. The Kolmogorov–Smirnov test was performed to examine whether the pRNFL thickness measurements were normally distributed. Univariate and multivariable linear regression models were constructed to examine the associations of pRNFL thickness with systemic variables and ocular parameters. Variables with a *P* value of less than 0.10 in univariate analyses and variables of scientific importance were included in multiple linear regression models. An additional linear regression model was then established to evaluate the independent effect of pRNFL thickness (independent variables) on the CSHQ score (dependent variable), adjusting for potential confounders such as gender, BMI, and refractive error. The *P* values less than 0.05 were considered statistically significant.

## 3. Results

Table 1 compares the characteristics between participants with and without pRNFL thickness data in grade 7 students. In general, there were no significant differences in terms of the distributions in gender, systematic, and ocular factors between those with and without pRNFL thickness data. Only significant difference in systolic BP was observed (*P*=0.01). The pRNFL thickness data were normally distributed (kurtosis = 0.8, skewness = 7.0, *P* for Kolmogorov–Smirnov test = 0.18). The mean pRNFL thickness was 106.8 ± 10.7 *μ*m among the 1063 participants.

**Figure 1:**
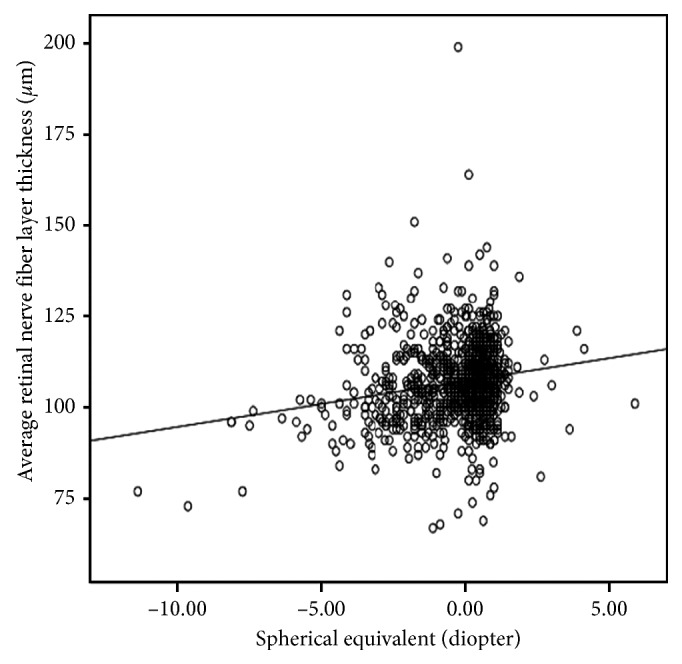
Scattergram on the relationship of mean peripapillary retinal nerve fiber layer thickness with spherical equivalent.

**Figure 2:**
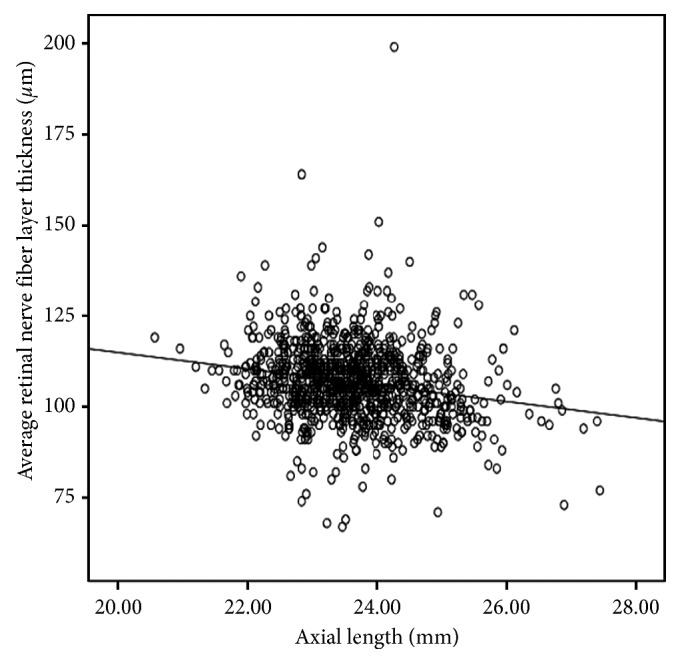
Scattergram on the relationship of mean peripapillary retinal nerve fiber layer thickness with axial length.

The associations of SE and AL with pRNFL thickness are plotted in Figures 1 and 2. There was an increasing trend of SE and a decreasing trend of AL with pRNFL thickness. Univariate and multivariate linear regression models were then constructed to explore the systemic and ocular predictors for pRNFL thickness in the 1063 students with SD-OCT data. In univariate comparisons, gender, refractive error, ACD, CP, and AL were found to be potential predictors for pRNFL thickness (all *P* < 0.10). Other factor such as BMI, BP, IOP, and birth weight were not significant associates (all *P* > 0.10). In multivariate analysis, gender and refractive error remained significant. Each diopter of SE increase was associated with a 0.75 *μ*m increase in pRNFL thickness. Girls had an increased mean pRNFL thickness compared with boys with a mean difference of 1.65 *μ*m observed (Table 2).

The association between pRNFL thickness and CSHQ score was assessed in a separate linear regression model with pRNFL thickness as the independent variable and the CSHQ score as the dependent variable. We found that every 10 *μ*m increase in pRNFL thickness was significantly associated with a 0.5 reduction in CSHQ score (better sleep quality) in both gender-adjusted and multivariate-adjusted models (Table 3). No significant associations were found between six sectors of pRNFL and CSHQ score (all *P* > 0.05). With regard to the different domains of the CSHQ, the presence of sleep anxiety (*P*=0.02) and sleep-disordered breathing (*P*=0.01) was found to be significantly related to reduced pRNFL thickness measurements.

## 4. Discussion

Our study reported that the mean pRNFL thickness as measured by SD-OCT was about 106.8 *μ*m in grade 7 Chinese students, which was greater than the estimates reported in other studies on Chinese students of similar ages [27]. More myopic refractive error was the major ocular determinant of decreased pRNFL thickness. In addition, adolescents with reduced pRNFL thickness tended to have a worse sleep quality.

The population-based estimate of mean pRNFL thickness in this study could be compared with the measurements reported in other studies, especially in younger generations. To the best of our knowledge, our study was most comparable to the Anyang Childhood Eye Study on Chinese students of similar ages residing in central China [27]. The Anyang Childhood Eye Study reported that the mean pRNFL thickness as assessed by the iVue OCT was 103.8 ± 9.0 *μ*m [27], which seems to be lower compared with the estimate in our study. The possible reason for the disparities in the mean pRNFL thickness could be explained by the lower prevalence of myopia in our study compared with that reported in the Anyang Childhood Eye Study [28] considering that myopic refractive error is a major determinant for a decreased pRNFL thickness of the eye. Other two population-based studies reported the mean pRNFL thickness in children and adolescents of a wider age range. The mean pRNFL thickness was observed to be 107 *μ*m in students aged 6 to 17 years in Shanghai [29] and 101 *μ*m in students aged 6 to 21 years in Inner Mongolia [30]. Other population-based studies on Chinese adults have also reported the mean pRNFL thickness estimates. In the Beijing Eye Study on Chinese adults aged 50 years or older, the mean pRNFL thickness measured by the iVue spectral-domain OCT was 103 *μ*m [31]. In Chinese living outside of China, the mean RNLF thickness as measured by the Cirrus OCT was 97.6 *μ*m in the Singapore Chinese Eye Study in people aged over 40 years [32]. It seems that there were no clear and consistent patterns in the age distributions of pRNFL thickness among Chinese populations. However, when comparing these estimates in different studies, methodological variations such as the sampling frames and the OCT machines used for measuring pRNFL thickness should be taken into consideration. For example, we have been aware of the differences in pRNFL thickness readings measured by different OCT systems. However, to the best of our knowledge, no studies have systematically assessed the impact of various OCT systems on pRNFL thickness readings. Thus, we cannot determine whether our study overestimated or underestimated the pRNFL thickness measurement compared with other studies.

With regards to the determinants of pRNFL thickness, we found that participants with more myopic refractive error tended to have thinner pRNFLs, which is consistent with most previous studies [28, 30, 32]. Myopia development together with axial elongation of the eyeball is known to result in pathologic changes in the retina such as the parapapillary gamma zone, while the axial elongation-associated changes were rarely seen in macular region [33, 34]. From a geometric perspective, the parapapillary surface area might be related to the thinning of the pRNFL in the parapapillary region in eyes with longer ALs, which may explain the associations of refractive error with pRNFL thickness [30]. Gender was found to be another determinant of pRNFL thickness in our study with girls having a thicker pRNFL than boys. Previous studies have reported conflicting results regarding the effect of gender on pRNFL thickness [14, 15, 35–38]. In this study, even after adjusting for refractive errors in multivariate analysis, girls still had a thicker pRNFL than boys. Further studies are warranted to elucidate whether women have more optic nerve fibers than men.

Another interesting finding of the study is that pRNFL thinning was related to a worse sleep quality in participants. The biological mechanisms of pRNFL thickness reduction in individuals with a worse sleep quality remain unclear. Studies on adults have consistently found that pRNFL thickness is lower in patients with obstructive sleep apnea syndrome. Disordered sleep or reduction in sleep quality may serve as a preclinical stage of obstructive sleep apnea in children and adolescents. Obstructive sleep apnea is related to a series of events such as transient hypoxemia and temporary elevation of artery resistance [19]. These abnormal changes may further result in apoptosis of retinal ganglion cells, which is associated with pRNFL thinning. However, our study indicated that every 10 *μ*m increase in pRNFL thickness was associated with a 0.5 reduction in CSHQ scores and whether the differences is of clinical importance remains unclear since we cannot determine whether this observed difference in CSHQ scores is related to an increased risk of obstructive sleep apnea syndrome. While the exact biological mechanisms linking thinner pRNFLs to a worse sleep quality in teenagers warrants further clarification, the observation might have important public health implications. Nowadays, sleep disorders are increasingly common health problems when people grow old. Extensive evidence has shown that worse sleep quality in adolescents might forebode sleep disturbances such as insomnia, sleep apnea, and restless legs syndrome in later life [39]. Our study provided a potential ocular marker for assessing sleep quality based on a noninvasive and easily accessible observation using SD-OCT devices. The findings suggest that assessing pRNFL thickness in early stages of life could be a new tool of risk stratifications for developing sleep disturbances in adulthood.

The strengths of our study included the population-based design with standardized protocols for measuring pRNFL thickness, which facilitated the interstudy comparisons among various populations. Additionally, we performed extensive systemic and ocular examinations on the participants, which allowed us to examine multiple potential determinants. Our study also had some limitations. First, although our results indicated that there were no significant differences between those with and without pRNFL thickness data, selection bias might still have occurred as we did not perform the SD-OCT measurement on the whole cohort due to limited resource in manpower. Second, whether the study findings could be extrapolated to Chinese students in other parts of China is uncertain considering that our site is located in a small county in Southwestern China. Third, pRNFL thickness measured by SD-OCT did not correct for magnification, and inaccuracy in the readings might exist. Further studies should consider magnificent error due to AL first, even though AL of the sample was not widely spread.

In conclusion, our study conducted in the southwestern part of China provided normative data on pRNFL thickness in grade 7 school students. More myopic refractive error is the major ocular determinant of decreased pRNFL thickness. In addition, students with reduced pRNFL thickness tended to have a worse sleep quality. Our results underscore the need for the establishment of more normative databases on pRNFL thickness among younger generations, which may in turn improve the sensitivity and specificity of the detection of glaucoma and pRNFL-related systemic diseases. Furthermore, our study also highlighted that evaluation of pRNFL thickness in teenagers may serve as a tool for predicting sleep quality.

## Figures and Tables

**Table 1:** Characteristics of participants with and without OCT scans.

	With OCT data (*n* = 1063)	Without OCT data (*n* = 1283)	*P* value
Sex, girls, no. (%)	524 (49.30)	609 (47.50)	0.38
Body mass index (kg/m^2^)	19.05 ± 2.87	18.87 ± 2.62	0.11
Systolic blood pressure (mmHg)	105.70 ± 12.68	106.99 ± 11.11	0.01
Diastolic blood pressure (mmHg)	66.62 ± 10.30	66.37 ± 8.89	0.53
Birth weight (kg)	2.67 ± 0.87	2.63 ± 0.88	0.21
Spherical equivalent (diopter)	−0.28 ± 1.59	−0.25 ± 1.34	0.12
Intraocular pressure (mmHg)	16.82 ± 3.32	16.68 ± 2.86	0.29
Axial length (mm)	23.55 ± 0.94	23.50 ± 0.86	0.07
Anterior chamber depth (mm)	3.08 ± 0.25	3.07 ± 0.26	0.10
Corneal power (diopter)	43.38 ± 1.36	43.33 ± 1.41	0.41

**Table 2:** Univariate and multivariable analyses on the predictors of peripapillary retinal nerve fiber layer thickness.

	Univariate analysis			Multivariable analysis		
	Beta	95% confidence interval	*P*	Beta	95% confidence interval	*P*
Sex, girls vs. boys	1.67	0.34, 3.00	0.01	1.65	0.22, 3.07	0.01
Body mass index, per kg/m^2^ increase	−0.03	−0.26, 0.20	0.80		—	
Systolic blood pressure, per mmHg increase	−0.02	−0.07, 0.04	0.55		—	
Diastolic blood pressure, per mmHg increase	0.02	−0.05, 0.08	0.63		—	
Birth weight, per kg increase	−0.02	−1.63, 1.60	0.98		—	
Spherical equivalent, per diopter increase	1.26	0.83, 1.68	<0.001	0.75	0.15, 1.35	<0.001
Intraocular pressure, per mmHg increase	−0.12	−0.34, 0.09	0.88		—	
Axial length, per mm increase	−2.25	−2.95, −1.55	<0.001		—	
Anterior chamber depth, per mm increase	−3.11	−5.74, −0.47	0.02	1.96	−1.45, 5.38	0.24
Corneal power, per diopter increase	0.48	0, 0.97	0.05	0.21	−0.61, 1.03	0.66

**Table 3:** Peripapillary retinal nerve fiber layer thickness as a predictor of the Children's Sleep Habits Questionnaire scores.

	Gender-adjusted		Multivariable-adjusted^*∗*^	
	Beta (95% confidence interval)	*P*	Beta (95% confidence interval)	*P*
Per 10 *μ*m increase in peripapillary retinal nerve fiber layer thickness	−0.5 (−0.9, −0.01)	0.04	−0.05 (−0.10, −0.01)	0.02

^*∗*^Adjusted for gender, body mass index, and refractive error.

## Data Availability

The data used to support the findings of this study are available from the corresponding author upon request.
